# Efficacy of repetitive transcranial magnetic stimulation for mild postpartum depression and its association with changes in plasma neuropeptide levels: a prospective, non-randomized controlled study

**DOI:** 10.3389/fpsyt.2026.1867202

**Published:** 2026-08-06

**Authors:** Meihong Huang, Donghua Shi, Jingling Ni, Qingxiao Shi, Ronghai Xu

**Affiliations:** 1 Department of Obstetrics, Jinjiang Municipal Hospital (Shanghai Sixth People’s Hospital Fujian), Jinjiang, Fujian, China; 2 Department of Neurology, Jinjiang Municipal Hospital (Shanghai Sixth People’s Hospital Fujian), Jinjiang, Fujian, China; 3 Department of Pharmacy, Jinjiang Municipal Hospital (Shanghai Sixth People’s Hospital Fujian), Jinjiang, Fujian, China

**Keywords:** brain-derived neurotrophic factor, neuropeptide, neuropeptide Y, oxytocin, postpartum depression, repetitive transcranial magnetic stimulation, substance P

## Abstract

**Objective:**

To evaluate the efficacy of high-frequency rTMS targeting the left DLPFC in mild postpartum depression (PPD) and its association with plasma neuropeptide levels (BDNF, NPY, SP, and oxytocin).

**Methods:**

In this prospective, non-randomized controlled study, 120 women with mild PPD received rTMS plus standard care (n = 60; 10 Hz, left DLPFC, 20 sessions over 4 weeks) or standard care alone (n = 60). HAMD-17 and EPDS scores and plasma BDNF, NPY, SP, and OXT (ELISA) were assessed at baseline and weeks 2, 4, and 8. Longitudinal between-group effects were analyzed using linear mixed-effects models, with neuropeptide comparisons corrected by the Benjamini-Hochberg false discovery rate (FDR).

**Results:**

Baseline characteristics were largely comparable, except for breastfeeding pattern (P = 0.012). At week 4, the rTMS group had lower HAMD-17 (6.55 vs. 9.30; P < 0.001) and EPDS scores (6.15 vs. 9.27; P < 0.001), with higher response (58.3% vs. 3.3%) and remission rates (71.7% vs. 15.0%; both P < 0.001), although controls also improved. rTMS-treated patients showed elevated plasma BDNF (27.30 vs. 20.27 ng/mL), NPY (114.60 vs. 98.63 pg/mL), and OXT (181.25 vs. 163.00 pg/mL) and reduced SP (52.69 vs. 62.27 pg/mL) at week 4 (all P ≤ 0.009; all surviving FDR correction). The OXT difference persisted after adjustment for baseline OXT and breastfeeding. Within-group neuropeptide changes did not correlate with individual symptom change. Benefits persisted at 8 weeks; adverse events were mild and self-limiting.

**Conclusions:**

In this non-randomized study without a sham control, rTMS added to standard care was associated with greater improvement in mild PPD than standard care alone, sustained through 8 weeks. The concurrent modulation of BDNF, NPY, SP, and OXT represents an exploratory association rather than an established mechanism. These preliminary findings require confirmation in multicenter, randomized, sham-controlled trials.

## 1 Introduction

Postpartum depression (PPD) affects approximately 10%–20% of women within the first year following delivery and constitutes the most common complication of childbirth (1, 2). The disorder manifests as persistent sadness, anhedonia, anxiety, sleep disturbance, and impaired maternal-infant bonding, with downstream consequences for child cognitive, emotional, and social development (3, 4). Despite its high prevalence and significant burden, treatment options for PPD remain constrained. Pharmacotherapy with selective serotonin reuptake inhibitors (SSRIs) remains the mainstay of treatment; however, maternal concerns regarding psychotropic exposure during lactation frequently limit adherence (5). Psychotherapy, while effective, demands sustained therapeutic engagement that many postpartum women find logistically challenging. These limitations underscore the need for novel, non-pharmacological interventions that can be delivered efficiently and with minimal systemic side effects.

Repetitive transcranial magnetic stimulation (rTMS) is a non-invasive neuromodulatory technique that utilizes time-varying magnetic fields to induce focused electrical currents within cortical tissue (6). High-frequency rTMS (≥5 Hz) applied to the left dorsolateral prefrontal cortex (DLPFC) has received regulatory approval for treatment-resistant major depressive disorder (MDD) and has demonstrated robust antidepressant efficacy across multiple randomized controlled trials and meta-analyses (7, 8). Several preliminary studies have extended these findings to postpartum populations, reporting favorable outcomes with acceptable safety profiles (9). A meta-analysis of randomized controlled trials evaluating rTMS for peripartum depression found significant reductions in depressive symptom severity compared with sham stimulation (10). Nevertheless, the existing evidence base remains limited by small sample sizes, heterogeneous study designs, and a paucity of mechanistic investigations.

The neurobiological mechanisms through which rTMS exerts its antidepressant effects are incompletely understood but are thought to involve neuroplasticity-related pathways. Brain-derived neurotrophic factor (BDNF) is a key mediator of synaptic plasticity and neuronal survival, and peripheral BDNF levels are consistently lower in individuals with MDD than in healthy controls (11). Several studies have demonstrated that rTMS can increase serum BDNF levels in patients with depression, although results remain mixed (12, 13). Beyond BDNF, neuropeptides such as neuropeptide Y (NPY), substance P (SP), and oxytocin (OXT) have been implicated in the pathophysiology of PPD. NPY functions as an endogenous anxiolytic and stress-resilience factor, with reduced plasma concentrations observed in PPD (14). SP, a tachykinin neuropeptide that activates neurokinin-1 receptors, is elevated in depressive states and modulates stress and inflammatory responses (15). OXT, widely recognized for its role in maternal bonding and social behavior, has been linked to PPD risk through diminished plasma concentrations during the peripartum period (16, 17).

Despite the therapeutic promise of rTMS and the biological plausibility of neuropeptide involvement in PPD, no prior study has systematically evaluated the effects of rTMS on a comprehensive panel of neuropeptides in postpartum women. This study was designed to address this gap by investigating the clinical efficacy of high-frequency rTMS for mild PPD and its association with temporal changes in plasma BDNF, NPY, SP, and OXT levels.

## 2 Methods

### 2.1 Study design and participants

This prospective, controlled clinical study was conducted at Jinjiang Municipal Hospital, Fujian, China, between September 2023 and August 2024. The study protocol was approved by the Institutional Ethics Committee of Jiniang Municipal Hospital (Shanghai sixth People’s Hospital Fujian) (approval number: jjsyyyxll2023060) and was conducted in accordance with the Declaration of Helsinki. Written informed consent was obtained from all participants.

During the study period, 2,000 postpartum women were systematically screened using the Edinburgh Postnatal Depression Scale (EPDS) within 2–8 weeks following delivery. Among these, 248 women received a diagnosis of PPD (EPDS ≥ 10), representing a prevalence of 12.4%.

Inclusion criteria were: (1) age 20–40 years; (2) singleton live birth; (3) EPDS score of 10–16 (mild depression range)20; (4) 17-item Hamilton Depression Rating Scale (HAMD-17) score of 8–17; (5) within 2–8 weeks postpartum; and (6) willingness to complete the full study protocol. Exclusion criteria comprised: (1) moderate to severe PPD (EPDS > 16 or HAMD-17 > 17); (2) history of bipolar disorder, psychotic disorders, or substance use disorders; (3) current use of psychotropic medications; (4) contraindications to rTMS (metallic implants, history of epilepsy, intracranial lesions); (5) severe obstetric complications or major medical illness; and (6) active suicidal ideation (HAMD-17 item 3 score ≥ 3).

**Figure 1:**
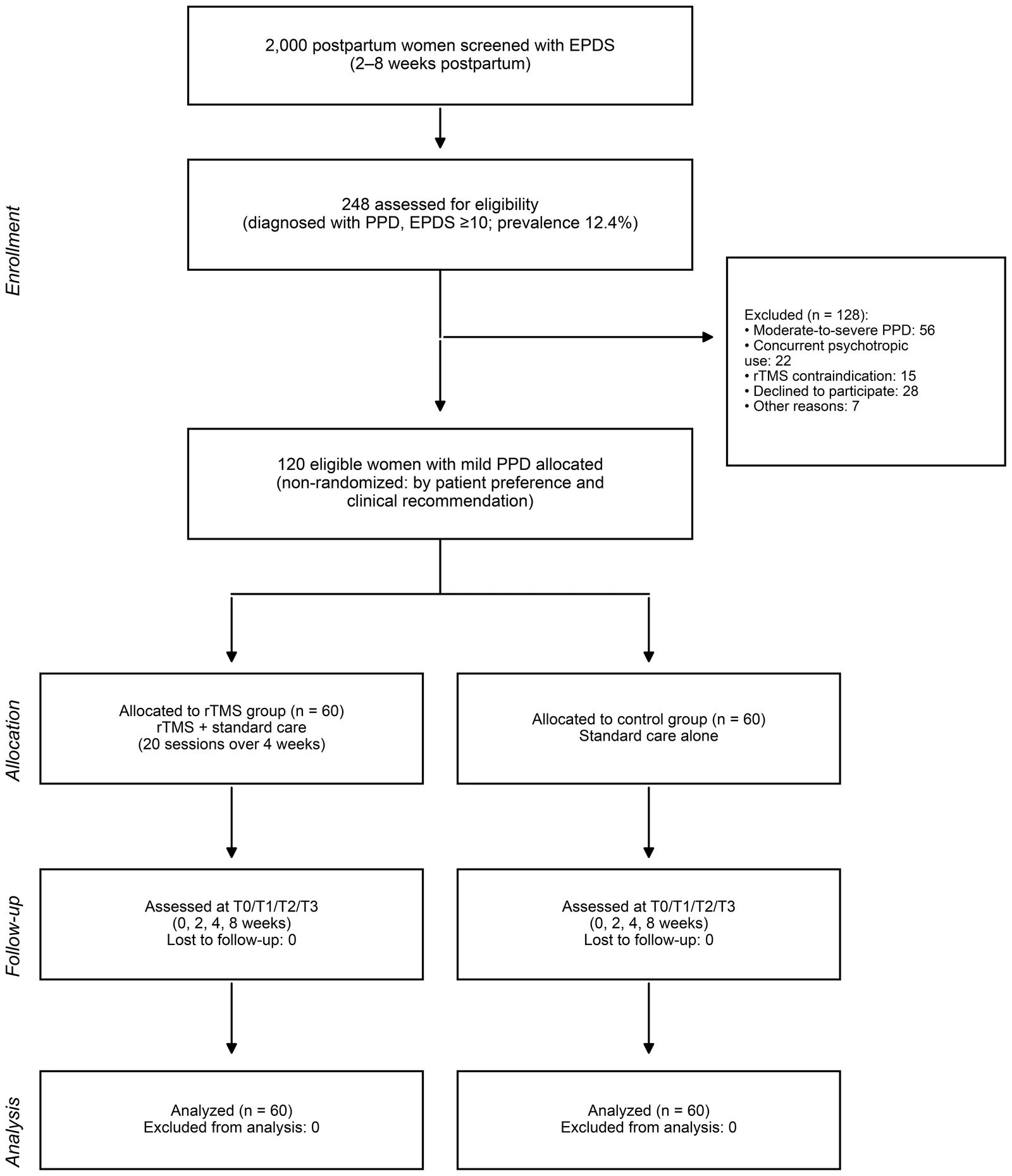
CONSORT-style flow diagram of participant screening, enrollment, allocation, follow-up, and analysis. Of 2,000 postpartum women screened with the Edinburgh Postnatal Depression Scale (EPDS) at 2–8 weeks postpartum, 248 were assessed for eligibility (diagnosed with postpartum depression [PPD]; EPDS >=10; prevalence 12.4%). After 128 exclusions (56 moderate-to-severe depression, 22 concurrent psychotropic use, 15 rTMS contraindications, 28 declining participation, 7 other), 120 women with mild PPD were allocated, on the basis of patient preference and clinical recommendation (non-randomized), to the rTMS group (n = 60; rTMS plus standard care) or the control group (n = 60; standard care alone). Both groups were assessed at baseline (T0) and weeks 2 (T1), 4 (T2), and 8 (T3); all 120 participants completed the protocol with no loss to follow-up and were included in the analysis.

Of the 248 women diagnosed with PPD, 128 were excluded (56 for moderate-to-severe depression, 22 for concurrent psychotropic medication use, 15 for rTMS contraindications, 28 for declining participation, and 7 for other reasons). The remaining 120 eligible participants were allocated into two groups based on patient preference and clinical recommendation: the rTMS group (n = 60), receiving rTMS combined with standard care, and the control group (n = 60), receiving standard care alone. The full participant screening, enrollment, and follow-up workflow is summarized in **Figure 1**. All 120 enrolled participants completed the study without dropout or loss to follow-up.

### 2.2 Intervention protocol

Participants in the rTMS group underwent high-frequency rTMS delivered by a certified technician using a MagPro R30 stimulator (MagVenture A/S, Farum, Denmark) equipped with a 70-mm figure-of-eight coil. The stimulation protocol was as follows: frequency, 10 Hz; intensity, 120% of resting motor threshold; stimulation site, left DLPFC (identified using the 5-cm rule anterior to the motor cortex hand area); pulse train, 4-second trains with a 26-second inter-train interval; total pulses per session, 3,000; session duration, approximately 37.5 minutes. Treatment was administered 5 days per week for 4 consecutive weeks, totaling 20 sessions.

Standard care for both groups comprised structured health education on postpartum mental health, supportive psychological counseling (one 30-minute session per week for 4 weeks), and routine postpartum follow-up care. No pharmacological antidepressant therapy was initiated in either group during the study period.

### 2.3 Clinical assessments

Clinical evaluations were performed at four time points: baseline (T0), week 2 (T1, midpoint of intervention), week 4 (T2, end of intervention), and week 8 (T3, follow-up). The primary outcome measures were the HAMD-17 and EPDS scores. All assessments were conducted by two trained psychiatrists who were not involved in delivering the rTMS intervention.

Treatment response was defined as a reduction of ≥50% from baseline in the HAMD-17 total score. Remission was defined as a HAMD-17 score of ≤7 at the assessment time point.

### 2.4 Plasma neuropeptide measurement

Fasting venous blood samples (5 mL) were collected from the antecubital vein between 7:00 and 9:00 AM at each time point (T0, T1, T2, T3). Samples were centrifuged at 3,000 rpm for 15 minutes within 30 minutes of collection, and plasma aliquots were stored at −80 °C until batch analysis. Plasma concentrations of BDNF, NPY, SP, and OXT were quantified using commercially available enzyme-linked immunosorbent assay (ELISA) kits (R&D Systems, Minneapolis, MN, USA) following the manufacturer’s protocols. Intra-assay and inter-assay coefficients of variation were maintained below 8% and 12%, respectively, for all analytes.

### 2.5 Statistical analysis

Statistical analyses were performed using SPSS version 26.0 (IBM Corp., Armonk, NY, USA) and Python 3.11 (statsmodels 0.14). Continuous variables were expressed as mean ± standard deviation (SD) and compared between groups using independent-samples t-tests; categorical variables were summarized as frequencies and percentages and compared using the chi-square test or Fisher exact test as appropriate. The primary longitudinal analyses of clinical scores and plasma neuropeptide levels were conducted using linear mixed-effects models, with group, time, and their interaction as fixed effects and a random intercept for each participant to account for the repeated measurements; the group × time interaction tested whether the temporal trajectory differed between groups. Repeated-measures analysis of variance (ANOVA) with Bonferroni-corrected *post-hoc* comparisons was retained as a secondary, consistency analysis. Because four neuropeptides were each compared between groups at three post-baseline time points, between-group comparisons were corrected for multiple testing using the Benjamini-Hochberg false discovery rate (FDR); both raw and FDR-adjusted P values are reported. Given the established influence of breastfeeding on oxytocin and the baseline imbalance in feeding pattern, the between-group difference in plasma OXT was additionally examined using analysis of covariance (ANCOVA) adjusted for baseline OXT and breastfeeding pattern. Associations between changes in clinical scores and changes in neuropeptide levels were assessed using Pearson correlation and are reported as exploratory, uncorrected analyses. A *post-hoc* power evaluation indicated that 60 participants per group provided approximately 80% power (two-sided α = 0.05) to detect a between-group standardized effect size of d ≈ 0.52, so the study was adequately powered for the primary clinical comparison. All tests were two-tailed, and P < 0.05 was considered statistically significant.

## 3 Results

### 3.1 Baseline characteristics

The demographic and clinical characteristics of the 120 participants at baseline are summarized in **Table 1**. The two groups were comparable in age, body mass index, gestational age at delivery, parity, delivery mode, education level, and days postpartum at enrollment (all P > 0.05). Breastfeeding pattern, however, differed significantly between groups (P = 0.012), with a higher proportion of formula feeding in the rTMS group (33.3% vs 11.7%); this imbalance was subsequently accounted for in the covariate-adjusted analysis of plasma OXT (see Section 3.3). Baseline HAMD-17 scores (rTMS: 13.37 ± 2.25 vs control: 12.80 ± 2.21; t = 1.393, P = 0.166) and EPDS scores (rTMS: 12.37 ± 1.46 vs control: 12.68 ± 1.47; t = −1.185, P = 0.239) did not differ significantly between groups. Baseline plasma neuropeptide levels were also comparable: BDNF (19.78 ± 4.38 vs 18.93 ± 4.35 ng/mL; P = 0.284), NPY (94.24 ± 19.78 vs 92.40 ± 18.86 pg/mL; P = 0.602), SP (69.17 ± 11.10 vs 68.61 ± 13.09 pg/mL; P = 0.799), and OXT (152.31 ± 29.64 vs 153.84 ± 29.89 pg/mL; P = 0.780). .

**Table 1:** Baseline demographic and clinical characteristics of study participants.

Variable	rTMS group (n = 60)	Control group (n = 60)	t/χ²	P
Age (years)	27.90 ± 3.51	28.82 ± 3.70	1.393	0.166
BMI (kg/m²)	24.49 ± 2.81	24.41 ± 2.21	0.177	0.860
Gestational weeks	39.21 ± 1.27	39.07 ± 1.26	0.592	0.555
Postpartum days	29.60 ± 11.75	29.95 ± 13.84	0.149	0.882
Parity				
Primiparous	33 (55.0%)	33 (55.0%)		0.854
Multiparous	27 (45.0%)	27 (45.0%)		
Delivery mode				
Vaginal	34 (56.7%)	40 (66.7%)	1.270	0.260
Cesarean	26 (43.3%)	20 (33.3%)		
Breastfeeding				
Exclusive	24 (40.0%)	31 (51.7%)		0.012
Mixed	16 (26.7%)	22 (36.7%)		
Formula	20 (33.3%)	7 (11.7%)		
HAMD-17 score	13.37 ± 2.25	12.80 ± 2.21	1.393	0.166
EPDS score	12.37 ± 1.46	12.68 ± 1.47	−1.185	0.239
BDNF (ng/mL)	19.78 ± 4.38	18.93 ± 4.35	1.076	0.284
NPY (pg/mL)	94.24 ± 19.78	92.40 ± 18.86	0.522	0.602
SP (pg/mL)	69.17 ± 11.10	68.61 ± 13.09	0.255	0.799
OXT (pg/mL)	152.31 ± 29.64	153.84 ± 29.89	−0.280	0.780

Data are presented as mean ± SD or n (%). BMI, body mass index; HAMD-17, 17-item Hamilton Depression Rating Scale; EPDS, Edinburgh Postnatal Depression Scale; BDNF, brain-derived neurotrophic factor; NPY, neuropeptide Y; SP, substance P; OXT, oxytocin.

### 3.2 Clinical outcomes

Both groups exhibited significant within-group reductions in HAMD-17 and EPDS scores over time (both P < 0.001 for repeated-measures ANOVA within each group). However, the magnitude of improvement was substantially greater in the rTMS group. Repeated-measures ANOVA revealed significant group × time interaction effects for both HAMD-17 (F = 38.72, P < 0.001) and EPDS (F = 45.18, P < 0.001). These interaction effects were confirmed by linear mixed-effects models (group × time: HAMD-17, F = 47.9, P < 0.001; EPDS, F = 54.9, P < 0.001), corroborating a steeper improvement trajectory in the rTMS group.

**Figure 2:**
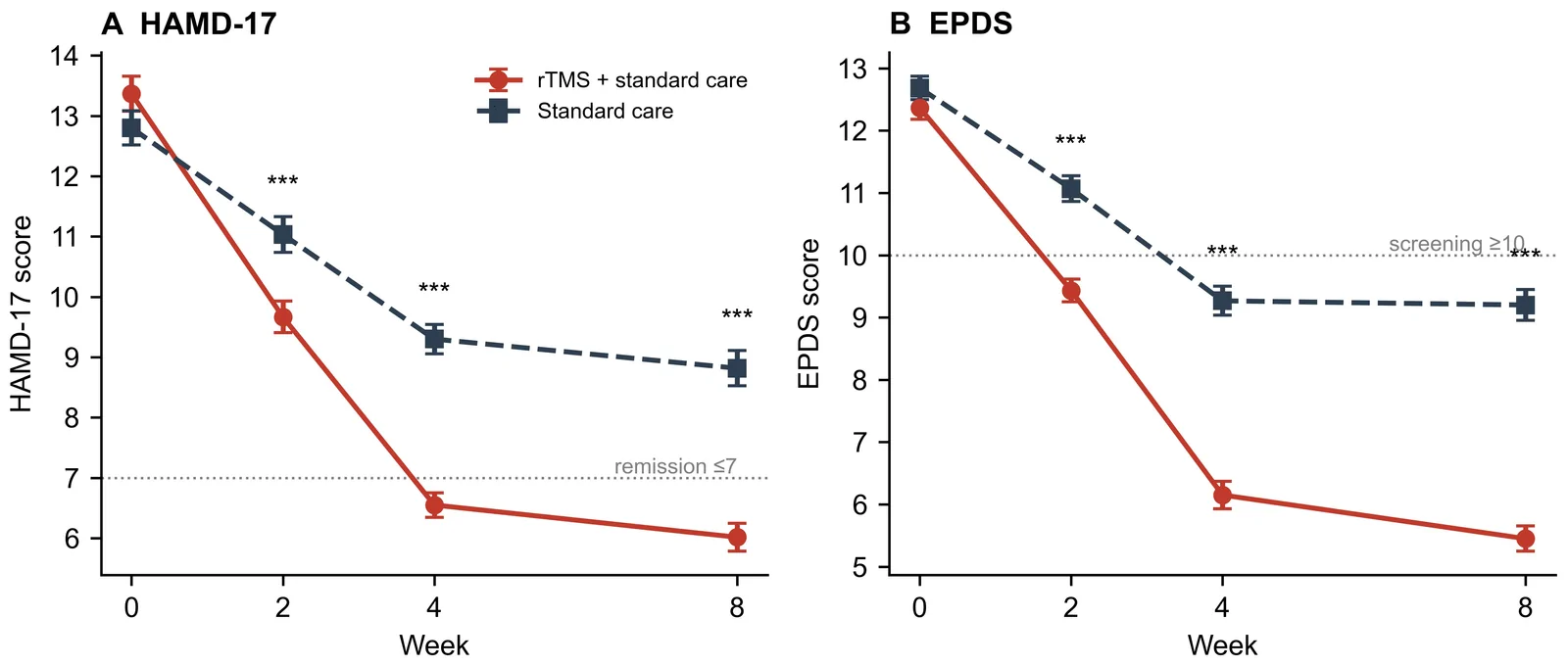
Trajectories of depressive symptom severity over the study period for **(A)** the 17-item Hamilton Depression Rating Scale (HAMD-17) and **(B)** the Edinburgh Postnatal Depression Scale (EPDS). Data are group mean +/- standard error of the mean (SEM; n = 60 per group). Solid red line, rTMS plus standard care; dashed dark line, standard care alone. Both groups improved over time, but the rTMS group showed a significantly steeper decline from week 2 onward (linear mixed-effects model group x time interaction: HAMD-17, F = 47.9; EPDS, F = 54.9; both P < 0.001). Asterisks denote between-group comparisons at each time point (independent-samples t-test): *P < 0.05, **P < 0.01, ***P < 0.001. Dotted reference lines indicate the HAMD-17 remission threshold (<=7) and the EPDS screening threshold (>=10).

By week 2, the rTMS group showed significantly lower HAMD-17 scores than the control group (9.67 ± 2.03 vs 11.03 ± 2.28; t = −3.470, P = 0.001). This divergence became more pronounced at week 4 (6.55 ± 1.56 vs 9.30 ± 1.90; t = −8.679, P < 0.001) and was maintained at the week 8 follow-up (6.02 ± 1.81 vs 8.82 ± 2.26; t = −7.496, P < 0.001). A parallel trajectory was observed for EPDS scores, with significant between-group differences emerging at week 2 (9.43 ± 1.43 vs 11.07 ± 1.58; t = −5.931, P < 0.001) and persisting through week 4 (6.15 ± 1.71 vs 9.27 ± 1.80; t = −9.728, P < 0.001) and week 8 (5.45 ± 1.58 vs 9.20 ± 1.90; t = −11.752, P < 0.001) (**Figure 2**).

**Figure 3:**
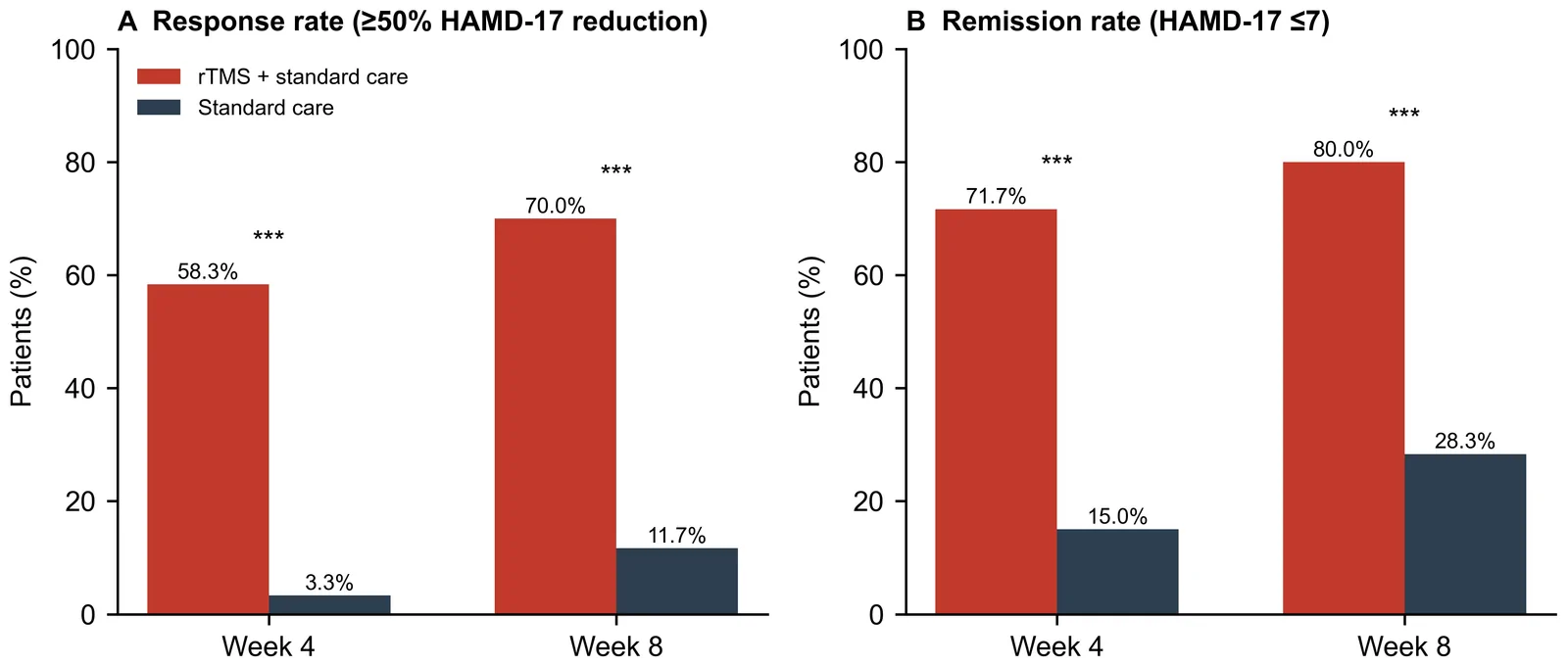
Treatment response and remission rates at weeks 4 and 8. **(A)** Response rate, defined as a >=50% reduction in HAMD-17 from baseline. **(B)** Remission rate, defined as a HAMD-17 score <=7. Red bars, rTMS plus standard care; dark bars, standard care alone (n = 60 per group); data labels show the percentage of patients in each group. Although the control group also improved over time, response and remission rates were significantly higher in the rTMS group at both time points. ***P < 0.001 (chi-square test).

The clinical response rate (≥50% HAMD-17 reduction) at week 4 was 58.3% (35/60) in the rTMS group compared with 3.3% (2/60) in the control group (P < 0.001). At week 8, these rates were 70.0% (42/60) and 11.7% (7/60), respectively (P < 0.001). The remission rate (HAMD-17 ≤ 7) at week 4 was 71.7% (43/60) in the rTMS group versus 15.0% (9/60) in the control group (P < 0.001), and at week 8, 80.0% (48/60) versus 28.3% (17/60) (P < 0.001) (**Figure 3**).

### 3.3 Changes in plasma neuropeptide levels

**Figure 4:**
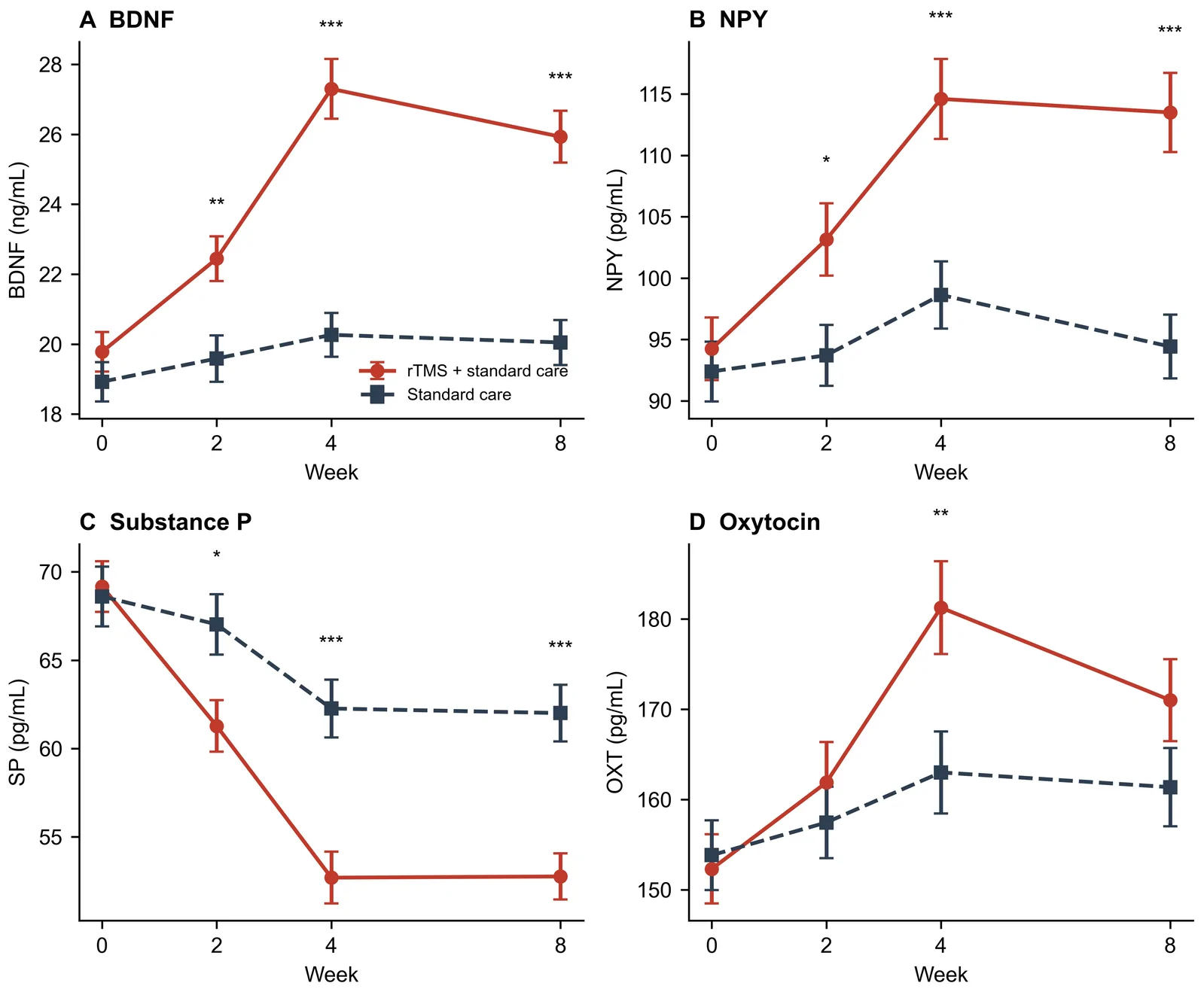
Trajectories of plasma neuropeptide concentrations over the study period: **(A)** brain-derived neurotrophic factor (BDNF), **(B)** neuropeptide Y (NPY), **(C)** substance P (SP), and **(D)** oxytocin (OXT). Data are group mean +/- SEM (n = 60 per group). Solid red line, rTMS plus standard care; dashed dark line, standard care alone. Linear mixed-effects models showed significant group x time interactions for all four analytes (BDNF, F = 73.1; NPY, F = 42.7; SP, F = 28.7; OXT, F = 18.0; all P < 0.001). Asterisks denote between-group comparisons at each time point that remained significant after Benjamini-Hochberg false-discovery-rate correction: *P < 0.05, **P < 0.01, ***P < 0.001. For OXT, the between-group difference reached significance only at week 4.

Repeated-measures ANOVA demonstrated significant group × time interaction effects for all four neuropeptides: BDNF, NPY, SP, and OXT (**Table 2**). The temporal trajectories of plasma neuropeptide concentrations in both groups are visualized in **Figure 4**. Linear mixed-effects models confirmed significant group × time interactions for all four analytes (BDNF, F = 73.1; NPY, F = 42.7; SP, F = 28.7; OXT, F = 18.0; all P < 0.001). All between-group neuropeptide differences that reached significance remained significant after Benjamini-Hochberg FDR correction; for OXT, the between-group difference was significant only at week 4 (FDR-adjusted P = 0.013), with no significant difference at weeks 2 or 8.

**Table 2:** Plasma neuropeptide levels at each time point.

Biomarker	Time	rTMS group (n = 60)	Control group (n = 60)	t	P
BDNF (ng/mL)	Baseline	19.78 ± 4.38	18.93 ± 4.35	1.076	0.284
	Week 2	22.44 ± 4.97	19.59 ± 5.13	3.094	0.002
	Week 4	27.30 ± 6.62	20.27 ± 4.88	6.623	<0.001
	Week 8	25.93 ± 5.75	20.05 ± 4.97	5.995	<0.001
NPY (pg/mL)	Baseline	94.24 ± 19.78	92.40 ± 18.86	0.522	0.602
	Week 2	103.15 ± 22.81	93.71 ± 19.28	2.448	0.016
	Week 4	114.60 ± 25.19	98.63 ± 21.20	3.757	<0.001
	Week 8	113.49 ± 24.91	94.42 ± 20.11	4.615	<0.001
SP (pg/mL)	Baseline	69.17 ± 11.10	68.61 ± 13.09	0.255	0.799
	Week 2	61.28 ± 11.30	67.03 ± 13.24	−2.559	0.012
	Week 4	52.69 ± 11.34	62.27 ± 12.67	−4.360	<0.001
	Week 8	52.76 ± 10.14	62.01 ± 12.41	−4.469	<0.001
OXT (pg/mL)	Baseline	152.31 ± 29.64	153.84 ± 29.89	−0.280	0.780
	Week 2	161.89 ± 34.79	157.46 ± 30.58	0.741	0.460
	Week 4	181.25 ± 39.88	163.00 ± 35.15	2.658	0.009
	Week 8	171.01 ± 35.17	161.37 ± 33.66	1.534	0.128

Data are presented as mean ± SD. BDNF, brain-derived neurotrophic factor; NPY, neuropeptide Y; SP, substance P; OXT, oxytocin.

Plasma BDNF levels in the rTMS group increased progressively from baseline (19.78 ± 4.38 ng/mL) to week 2 (22.44 ± 4.97 ng/mL) and peaked at week 4 (27.30 ± 6.62 ng/mL), with a modest decline by week 8 (25.93 ± 5.75 ng/mL) that remained significantly above baseline. Between-group comparisons revealed significantly higher BDNF levels in the rTMS group at week 2 (P = 0.002), week 4 (P < 0.001), and week 8 (P < 0.001). In contrast, BDNF levels in the control group exhibited only marginal fluctuation throughout the study period.

Plasma NPY levels followed a similar upward trajectory in the rTMS group, rising from 94.24 ± 19.78 pg/mL at baseline to 114.60 ± 25.19 pg/mL at week 4 and stabilizing at 113.49 ± 24.91 pg/mL at week 8. The between-group difference reached significance at week 2 (P = 0.016), week 4 (P < 0.001), and week 8 (P < 0.001).

Plasma SP levels declined significantly in the rTMS group, decreasing from 69.17 ± 11.10 pg/mL at baseline to 52.69 ± 11.34 pg/mL at week 4 and remaining reduced at 52.76 ± 10.14 pg/mL at week 8. Between-group comparisons showed significantly lower SP levels in the rTMS group at week 2 (P = 0.012), week 4 (P < 0.001), and week 8 (P < 0.001).

Plasma OXT levels rose from 152.31 ± 29.64 pg/mL at baseline to 181.25 ± 39.88 pg/mL at week 4 in the rTMS group, with a subsequent decline to 171.01 ± 35.17 pg/mL at week 8. A significant between-group difference was observed at week 4 (P = 0.009), although the difference at week 2 (P = 0.460) and week 8 (P = 0.128) did not reach statistical significance. Because breastfeeding pattern was imbalanced at baseline and strongly influences oxytocin, the week-4 between-group difference in OXT was re-examined using ANCOVA adjusted for baseline OXT and breastfeeding pattern; the difference remained significant (adjusted between-group difference 18.8 pg/mL, 95% CI 13.6-24.0, P < 0.001), indicating that the OXT finding was not attributable to the feeding imbalance alone.

### 3.4 Correlation analysis

In the rTMS group, Pearson correlation analysis was performed to evaluate associations between the magnitude of clinical improvement (change in HAMD-17 and EPDS from baseline to week 4) and changes in plasma neuropeptide levels over the same interval (**Table 3**). No individual correlation reached statistical significance, with r values ranging from −0.177 to 0.182 (all P > 0.05). Analogous analyses using EPDS change as the clinical variable yielded comparable results. Given the absence of significant individual-level correlations, the group-level neuropeptide changes are interpreted as exploratory associations rather than as direct mediators of symptom improvement.

**Table 3:** Pearson correlation between changes in clinical scores and neuropeptide levels (rTMS group, baseline to week 4).

Correlation pair	r	P	95% CI
ΔHAMD-17 vs ΔBDNF	0.109	0.408	−0.149, 0.353
ΔHAMD-17 vs ΔNPY	0.182	0.163	−0.076, 0.416
ΔHAMD-17 vs ΔSP	−0.158	0.229	−0.396, 0.100
ΔHAMD-17 vs ΔOXT	−0.177	0.175	−0.413, 0.081
ΔEPDS vs ΔBDNF	−0.106	0.419	−0.351, 0.153
ΔEPDS vs ΔNPY	−0.065	0.622	−0.317, 0.195
ΔEPDS vs ΔSP	0.046	0.728	−0.213, 0.299
ΔEPDS vs ΔOXT	0.043	0.745	−0.216, 0.297

Δ, Change from baseline to week 4. CI, confidence interval.

### 3.5 Adverse events

rTMS was generally well tolerated. In the rTMS group, 21 of 60 participants (35.0%) reported at least one adverse event, compared with 4 of 60 (6.7%) in the control group. The most frequently reported adverse events in the rTMS group were mild headache (10/60, 16.7%), scalp discomfort at the stimulation site (7/60, 11.7%), transient dizziness (3/60, 5.0%), and insomnia (1/60, 1.7%). All adverse events were grade 1 (mild), self-limiting, and did not necessitate treatment discontinuation or dose modification. No serious adverse events, seizures, or manic episodes were observed in either group throughout the study period (**Table 4**).

**Table 4:** Adverse events during the study period.

Adverse event	rTMS group (n = 60)	Control group (n = 60)	P
Any adverse event	21 (35.0%)	4 (6.7%)	<0.001
Mild headache	10 (16.7%)	1 (1.7%)	0.004
Scalp discomfort	7 (11.7%)	0 (0.0%)	0.013
Dizziness	3 (5.0%)	0 (0.0%)	0.244
Insomnia	1 (1.7%)	3 (5.0%)	0.619

Data are presented as n (%). Fisher exact test was used when expected cell count < 5.

## 4 Discussion

This prospective clinical study provides evidence that high-frequency rTMS applied to the left DLPFC, in conjunction with standard care, was associated with clinically meaningful and statistically significant improvement in depressive symptoms among women with mild PPD, with treatment benefits sustained through an 8-week follow-up. Furthermore, rTMS treatment was associated with concurrent modulation of plasma neuropeptide levels—specifically, increases in BDNF, NPY, and OXT, and decreases in SP—suggesting that the antidepressant action of rTMS in PPD may involve coordinated regulation of multiple neuropeptide systems. Because allocation was non-randomized and no sham comparator was used, however, these associations are interpreted cautiously and are not taken as evidence of a specific neuromodulatory mechanism.

The clinical outcomes observed in our study are consistent with, and extend, a growing body of evidence supporting the efficacy of rTMS in perinatal depression (9, 10, 18). The response rate of 58.3% and remission rate of 71.7% at the conclusion of the 4-week treatment course compare favorably with rates reported in prior open-label trials of rTMS for PPD, which have documented remission rates ranging from 66% to 90% (9, 18). These figures exceed the typical response rates observed in the general MDD population receiving rTMS, which approximate 50% for response and 25%–30% for remission (7). Several factors may explain this enhanced treatment response, including the relatively milder severity of depression in our cohort, the absence of treatment resistance as an inclusion criterion, and the neurobiological characteristics unique to the peripartum period, such as heightened neuroplasticity driven by hormonal flux (19, 20). Importantly, the control group also showed appreciable symptom reduction over time, consistent with the high rate of spontaneous improvement that characterizes mild PPD during the early postpartum period (29). The between-group contrast therefore reflects the incremental benefit of rTMS added to enhanced clinical contact above natural recovery, rather than the full magnitude of the observed improvement, and the response and remission rates should be interpreted in this light.

Notably, the therapeutic benefits of rTMS were evident as early as week 2 and continued to accrue through the 4-week treatment period, with sustained improvement at the 8-week follow-up assessment. This temporal profile suggests that rTMS initiates early neurobiological processes that consolidate over time, a pattern consistent with the neuroplasticity hypothesis of rTMS action (21). The sustained benefit at follow-up is particularly relevant for clinical practice, as it addresses concerns regarding the durability of rTMS effects in the absence of ongoing treatment.

The elevation of plasma BDNF levels following rTMS treatment represents a key finding of this investigation. BDNF occupies a central position in the neurotrophic hypothesis of depression, which posits that depressive disorders are characterized by impaired neuroplasticity and that effective antidepressant treatments restore neurotrophic signaling (22). Preclinical studies have demonstrated that rTMS increases BDNF mRNA expression in the hippocampus and cortex (23), and several clinical studies in MDD have reported rTMS-induced increases in peripheral BDNF levels (12, 24). A recent randomized case-control study by Ozkan et al. (13) demonstrated that 8-week rTMS monotherapy significantly elevated serum BDNF and glial cell line-derived neurotrophic factor in patients with MDD, accompanied by reductions in oxidative stress markers. Our findings extend these observations to the postpartum population and confirm that rTMS-induced BDNF elevation is a reproducible neurobiological correlate of the antidepressant response across diverse patient populations.

The increase in plasma NPY levels observed in the rTMS group provides novel insight into the neuropeptide mechanisms of rTMS. NPY is one of the most abundant peptides in the mammalian central nervous system and functions as a potent endogenous anxiolytic and anti-stress mediator (25). Reduced NPY levels have been documented in PPD and are inversely correlated with norepinephrine concentrations, suggesting a role for NPY in modulating stress-related neurotransmission (14). While preclinical data indicate that rTMS can modulate NPY expression in specific brain regions (23), clinical evidence linking rTMS to peripheral NPY changes has been lacking. Our finding that rTMS restores NPY levels in women with mild PPD represents, to our knowledge, the first clinical demonstration of this association, implicating NPY-mediated stress buffering as a component of the therapeutic mechanism.

The reduction in plasma SP levels following rTMS aligns with the role of SP in the neurobiology of depression and pain. SP acts through the neurokinin-1 (NK-1) receptor and has been linked to heightened stress responses, neuroinflammation, and depressive symptomatology (15). Elevated SP levels have been reported in PPD patients compared with non-depressed postpartum controls (14). Pharmacological antagonism of the NK-1 receptor has shown antidepressant potential in clinical trials, suggesting that downregulation of SP signaling constitutes a viable therapeutic target (26). The rTMS-associated decrease in SP observed in our study supports the hypothesis that neuromodulation may attenuate pro-inflammatory and stress-responsive neuropeptide pathways, complementing its direct cortical effects.

The transient increase in plasma OXT levels, reaching significance only at week 4, is noteworthy given the established connection between OXT and maternal behavior. Reduced OXT levels have been associated with PPD risk, impaired maternal-infant bonding, and heightened stress reactivity (16, 17). The stimulation of the left DLPFC may indirectly modulate hypothalamic OXT release through cortico-limbic-hypothalamic circuits (27). The attenuation of the OXT effect at the 8-week follow-up may reflect the cessation of rTMS stimulation, suggesting that OXT modulation may require ongoing or maintenance neuromodulatory input—a hypothesis that warrants investigation in future maintenance-protocol studies. Plasma oxytocin is additionally influenced by breastfeeding, mother-infant interaction, sleep, and hormonal fluctuation, and breastfeeding pattern was imbalanced between our groups; the week-4 between-group difference nonetheless persisted after adjustment for breastfeeding and baseline OXT, although residual confounding from these unmeasured behavioral factors cannot be excluded, and the OXT findings should be regarded as preliminary.

While group-level neuropeptide changes were statistically robust, individual-level correlations between the magnitude of clinical improvement and the degree of neuropeptide change were modest and did not reach statistical significance. This observation is not without precedent; Pan et al. (12) similarly reported significant group differences in BDNF following rTMS treatment for depression without significant individual-level correlations between biomarker and clinical changes. These findings suggest that the antidepressant effect of rTMS likely involves the convergent modulation of multiple neurobiological pathways, of which neuropeptide changes represent one component within a broader network of neuroplastic, neurotransmitter, and connectivity-related adaptations (21, 28). Moreover, because multiple neuropeptides were assessed at several time points, we applied FDR correction to the between-group comparisons; while the principal group-level differences survived correction, the nonsignificant individual correlations reinforce that these neuropeptide changes should be viewed as exploratory associations rather than validated mechanistic biomarkers.

Several methodological strengths of this study deserve mention. The prospective design, standardized rTMS protocol, and systematic assessment at four time points allow for robust longitudinal characterization of both clinical and biomarker trajectories. The inclusion of a comprehensive neuropeptide panel provides a more nuanced view of the neurobiological effects of rTMS than studies measuring a single biomarker. The recruitment of a well-characterized population of women with mild PPD, with balanced groups at baseline, supports the internal validity of the findings.

Several limitations must be acknowledged. First, allocation was non-randomized and based on patient preference and clinical recommendation, which introduces a substantial risk of selection bias; participants choosing rTMS may differ from controls in motivation, treatment expectation, psychosocial support, or healthcare engagement, and these factors could influence outcomes independently of the intervention. The causal interpretation of the treatment effect is therefore necessarily cautious. Second, no sham-rTMS arm was included. Because the rTMS group received an intensive device-based intervention with frequent clinician contact, placebo and expectancy effects cannot be excluded; the present design effectively compares an enhanced intervention plus routine care against routine care alone, rather than active neuromodulation against sham stimulation, which limits attribution of the antidepressant effect specifically to cortical stimulation. Third, the study enrolled women with mild PPD during the early postpartum period, when spontaneous symptom improvement is common; although the between-group difference exceeded the improvement seen with standard care alone, natural remission may still have contributed to the observed effect, and the response and remission rates should not be interpreted as attributable to rTMS alone. Fourth, breastfeeding pattern was imbalanced at baseline; although the OXT finding persisted after covariate adjustment, residual confounding from breastfeeding and related behavioral factors cannot be excluded. Fifth, peripheral neuropeptide concentrations are an imperfect proxy for central nervous system activity, and the group-level neuropeptide changes were not accompanied by significant individual-level correlations with symptom change, so they represent exploratory associations rather than established mechanisms. Sixth, the single-center design and the restriction to mild PPD limit generalizability; the findings cannot be extended to moderate-to-severe postpartum depression. Finally, the 8-week follow-up is insufficient to evaluate long-term durability. Adequately powered, multicenter, randomized, sham-controlled trials with longer follow-up are required to confirm these preliminary findings.

## 5 Conclusions

In this prospective but non-randomized study without a sham control, high-frequency rTMS targeting the left DLPFC added to standard postpartum care was associated with greater clinical improvement than standard care alone in women with mild PPD, with benefits sustained for at least 4 weeks after treatment completion, and was well tolerated. rTMS was accompanied by modulation of plasma neuropeptide profiles—increases in BDNF, NPY, and OXT and decreases in SP—but, in the absence of significant individual-level correlations with symptom change, these represent exploratory associations rather than established therapeutic mechanisms. Given the non-randomized design, the absence of a sham comparator, and the possible contribution of natural remission, these results should be regarded as preliminary and hypothesis-generating. Confirmation in multicenter, randomized, sham-controlled trials with larger samples and longer follow-up is required before rTMS can be recommended for the routine clinical management of mild PPD.

## Data Availability

The original contributions presented in the study are included in the article/supplementary material. Further inquiries can be directed to the corresponding author.
